# Novel Graphene Electrode for Retinal Implants: An *in vivo* Biocompatibility Study

**DOI:** 10.3389/fnins.2021.615256

**Published:** 2021-03-04

**Authors:** Diep Nguyen, Manon Valet, Julie Dégardin, Leyna Boucherit, Xavi Illa, Jose de la Cruz, Elena del Corro, Jessica Bousquet, Jose A. Garrido, Clément Hébert, Serge Picaud

**Affiliations:** ^1^INSERM, CNRS, Institut de la Vision, Sorbonne Université, Paris, France; ^2^Instituto de Microelectrónica de Barcelona, IMB-CNM (CSIC), Bellaterra, Spain; ^3^Centro de Investigación Biomédica en Red en Bioingeniería, Biomateriales y Nanomedicina, Madrid, Spain; ^4^Catalan Institute of Nanoscience and Nanotechnology, Barcelona, Spain; ^5^Catalan Institution for Research and Advanced Studies, Barcelona, Spain

**Keywords:** graphene, prosthesis, biocompatibility, retina, implant

## Abstract

Evaluating biocompatibility is a core essential step to introducing a new material as a candidate for brain-machine interfaces. Foreign body reactions often result in glial scars that can impede the performance of the interface. Having a high conductivity and large electrochemical window, graphene is a candidate material for electrical stimulation with retinal prosthesis. In this study, non-functional devices consisting of chemical vapor deposition (CVD) graphene embedded onto polyimide/SU-8 substrates were fabricated for a biocompatibility study. The devices were implanted beneath the retina of blind P23H rats. Implants were monitored by optical coherence tomography (OCT) and eye fundus which indicated a high stability *in vivo* up to 3 months before histology studies were done. Microglial reconstruction through confocal imaging illustrates that the presence of graphene on polyimide reduced the number of microglial cells in the retina compared to polyimide alone, thereby indicating a high biocompatibility. This study highlights an interesting approach to assess material biocompatibility in a tissue model of central nervous system, the retina, which is easily accessed optically and surgically.

## Introduction

Brain machine interfaces are emerging technologies to restore perception and action following different degenerative diseases and traumatic incidents (Slutzky, 2019). These interfaces can be used directly on the brain or in more peripheral locations of the central nervous systems. In this context, retinal prostheses have offered solutions to restore some useful vision in blind patients (da Cruz et al., 2016; Stingl et al., 2017; Palanker et al., 2020). The targeted vision impairment is due to retinitis pigmentosa and age-related macular degeneration that leads to the progressive loss of photoreceptors in the retina. Though there exists no cure, rehabilitation therapy through electrical stimulation at the retina has enabled late-stage patients to experience visual sensation including the ability to read albeit with a low visual acuity (Hornig et al., 2017; Hahn and Fine, 2018). The Argus II (Second Sight) (da Cruz et al., 2016) and the Alpha-AMS (Retina Implant AG) (Edwards et al., 2018) devices have received approval for market sale to patients with end stage retinitis pigmentosa by either the US FDA and European CE or only European CE, respectively. More recently, the Prima device was directly tested in patients with age-related macular degeneration due to the expected high visual acuity (Lorach et al., 2015; Palanker et al., 2020; Prévot et al., 2020). In all implant designs, the devices contain an array of electrodes either in the subretinal space (Alpha IMS, Prima) (Stingl et al., 2017; Palanker et al., 2020) or in the epiretinal position in the vitreous body (da Cruz et al., 2016). Electrical stimulation of the retina generates phosphenes (visual sensation) to patients who learn over time to associate them to form basic vision. However, current devices are unable to provide facial recognition, independent locomotion, and complex text reading. They require improvements to provide restored vision of higher resolution.

For retinal prostheses, as for most brain machine interfaces, conductive electrode materials with a high double layer capacitance are expected to offer improved spatial resolution (Joucla and Yvert, 2009). Carbon related materials and specifically graphene and graphene derivatives are currently being studied for their electrical properties as biosensors (Pumera et al., 2010) and neural interfacing (Kostarelos et al., 2017). As neural interfacing materials, graphene and their derivatives make up a class of electrode material that have advantageous properties for recording and stimulation. Graphene is a single-layered, transparent but visible, 2-D material that has a large electrochemical water window and high electron mobility (Ji et al., 2014). Graphene is mainly grown at high temperatures on metallic substrates such as copper in chemical vapor deposition (CVD) reactors, and can be wet transferred onto flexible polymer substrates (Lee et al., 2010). The high transparency of CVD graphene has already been exploited in acute cortical recordings simultaneously with calcium imaging (Kuzum et al., 2014) as well as measuring electroretinograms in non-human primates (Yin et al., 2018). Fabricating a 90% transparent device with graphene electrodes has demonstrated its capability to record brain signals during light dependent applications such as florescence microscopy and optogenetics (Park et al., 2014). For neural stimulation, flat CVD graphene is often considered a poor material lacking the desired charge capacity of metals. However, some have demonstrated its stimulation capabilities to active cortical neurons (Park et al., 2018). In addition, 3D nanostructures of graphene using porous templates (Fan et al., 2013; Li et al., 2013), graphene foam (Dong et al., 2012), and graphene mesh (Jiang and Fan, 2014) have demonstrated to maintain graphene material properties with increased electrochemical surface area for an improved charge storage capacity that could be beneficial for neural stimulation. Additionally, reduced graphene oxide generally made by redox treatment of graphite have already been fabricated to include porous structures intended for neural stimulation (Apollo et al., 2015; Lu et al., 2016).

However, despite having ideal electrical properties, the safety and biocompatibility of new materials must be evaluated before determining their relevance in stimulation applications. A host rejection to foreign material over time can render a device useless, causing complications and requiring additional surgery. For instances, gold electrodes were observed to have degraded within a few months below the retina (Chow et al., 2001). Various studies have provided evidence for the biocompatibility of graphene and graphene derivatives to many cell types such as cultured human and mouse fibroblast and various *in vivo* organs like rodent liver and lung showing high cell viability and survival rates despite some visual changes in histological results (Pinto et al., 2013). Because tissue reactions can be very cell specific, there are few studies evaluating the biocompatibility of graphene in the central nervous system. It has been previously shown that single layer graphene is capable of promoting an improved neurite growth and adhesion for retinal ganglion neurons compared to glass (Bendali et al., 2013) and that primary hippocampal neurons has a significantly better adherence to graphene compared to insulating polymers as well as CVD diamond in the absence of poly-L-lysine (Bourrier et al., 2019). However, no *in vivo* study has been completed to show a chronic biocompatibility of graphene in the ocular environment.

In this study, the retina was used to investigate an *in vivo* biocompatibility of graphene due its highly structured organization in layers, which enables the identification of toxic changes. The biocompatibility of graphene was examined in the ocular environment of the P23H rat, a model of retinitis pigmentosa. Soft and flexible subretinal implants with large CVD graphene surfaces were designed and adapted to test biocompatibility in rats. An *in vivo* monitoring of the implant was taken to assess the retinal state and the implant stability in the eye. Finally, a post mortem histological study was performed to quantify microglial cells of the retina, which are an excellent biomarker to monitor the reactive state (Polikov et al., 2005), and thus the biocompatibility of graphene compared to a control implant made of soft insulating polymers.

## Materials and Methods

### Microfabrication of Device

**FIGURE 1:**
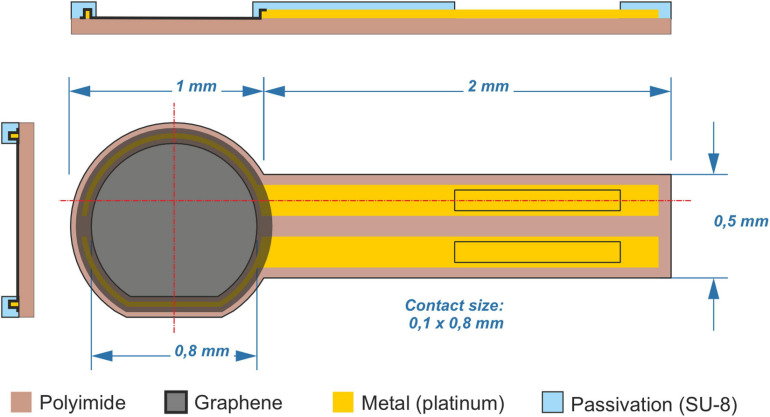
Graphene-based implants. Orthogonal schemes of fabricated implants showing the side and top views. Graphene and platinum lines are passivated between polyimide and SU-8. Control implants have the same scheme without the graphene layer, exposing instead the bottom polyimide layer.

Implants were designed to maximize the exposed area of graphene on the circular head to the tissue with the underlying metal contacts to validate the presence of graphene by a resistance measurement (Figure 1). They were fabricated using a 4-inch thermally oxidized Si wafer as a carrier support. A 10-μm thick polyimide layer (PI-2611, HD MicroSystems) was spin-coated and cured twice to obtain a substrate with a 20-μm thickness. A lift-off process using image reversal photoresist AZ5214 (Clariant GmbH, Germany) was used to define the metal tracks (Ti/Pt, 10/100 nm) that were deposited using an electron-beam vapor deposition system. Single layer graphene was grown by CVD and transferred onto the polyimide substrate using a wet chemical method as previously described (Blaschke et al., 2016; Hébert et al., 2017). Large graphene areas (1 mm in diameter) on top of the metal tracks were protected using a positive photoresist (HiPR 6512, FujiFilm) that was used as a mask for etching the rest of the graphene layer by means of an oxygen-based reactive ion etching (RIE). After removing the photoresist, the negative photoresist SU-8 (SU-8 2005, MicroChemCorp., United States) was spin-coated and defined to passivate the metal leads while defining the uncovered graphene area. To define the final geometry of the implants, a final photolithographic process using a thick positive photoresist (AZ9260, Clariant) was used to define the mask for structuring the 20-μm thick polyimide layer in a deep-RIE process. The wafer was then cleaned in isopropanol and rinsed with water before being peeled off from the silicon wafer.

In parallel, a second wafer was processed to fabricate the control devices. The process was identical as the one described above but without the implementation of the graphene layer.

### Subretinal Implantation

Twelve homozygous P23H rats were implanted with the fabricated implant devices: *N* = 7 with graphene and *N* = 5 without graphene as a sham. Seven non-implanted eyes were used for a retina-only control. Animals were enclosed in a controlled environment with a half-day dark/light cycle with nutrition *ad libitum*. All animal experiments were completed in accordance to the Charles Darwin No5 Ethics Committee in Animal Experimentation (agreement #15219) in the approved animal facilities associated to the Institut de la Vision (Sorbonne Université). All experimental work were performed according to the institutional policies on biosecurity and safety procedures. All animals were unilaterally implanted at 9-months of age. The short surgery consisted of placing the device in the subretinal space in the central region next to the optic nerve as previously described (Salzmann et al., 2006). Briefly, anesthesia was provided by an intraperitoneal injection of a ketamine-medetomidine mixture (40 mg kg^–1^ ketamine, 0.14 mg kg^–1^ medetomidine) following a 5% gaseous induction of isoflurane. An additional ocular anesthesia using oxybuprocaïn chlorohydrate eye drops was administered. Eye dilatation was obtained by application of tropicamide (0.5%) solution. A heating platform maintained body temperature at 37°C. A small sclerotomy was performed on the dorsal sclera tangential to the cornea. A gel of sodium chondroitin sulfate – sodium hyaluronate (Viscoat Alcon) was injected in the sclerotomy to generate a retinal detachment. The implant was then inserted below the detached retina in the subretinal space targeting an adjacent location to the optic disk. Observation under the surgical microscope confirmed the placement in the subretinal position using a plastic coverslip gently pressed on ophthalmic gel that was applied to the cornea. Subcutaneous injection of atipamezole (antisedan) was then used to reanimate the animal in a chamber maintained 37°C.

### *In vivo* Monitoring

*In vivo* monitoring of implant state consisted of eye fundus and optical coherence tomography (OCT) imaging done at 7, 15, 30, 60, and 90 days post implantation. Eye fundus images were taken on a Micron III and Micron IV machines (Pheonix Technology Group, Pleasanton, CA, United States) and OCT imaging was done on a Bioptigen OCT system (Leica microsystems). Animal anesthesia, eye dilation, and reanimation were performed in the same manner as explained in the implantation section. Immediately after the 90-day time point, the animals were euthanized by CO_2_ induction followed by cervical dislocation.

### Immunohistochemistry

Immediately after euthanasia, the implanted eye was removed and dissected. The cornea and lens were removed in phosphate buffer saline (PBS) solution (0.1 M, pH 7.4). A 3-mm biopsy punch in the eye cup was used to extract the implant between the retina and the sclera and choroid. This eye fragment was fixed by incubation overnight in paraformaldehyde solution (4% in PBS) at 4°C and then washed using PBS.

For immunolabelling, eye fragments were incubated in a blocking solution (10% bovine serum albumin (Sigma, France), 2% Triton X-100 (Sigma), 0.5% Tween 20 (Sigma, France) and 0.1 g L^–1^ Thimerosal (Sigma, France) in PBS for 1 h at room temperature. Afterward, a 3-day incubation at 4°C with slow stirring was done, followed by an incubation at room temperature for 2 h with primary antibodies in blocking solution. The antibodies used were polyclonal antibodies directed against Chicken anti Glial Fibrillary Acidic Protein (1:100, LifeSpan Biosciences, WA, United States), Rabbit anti Iba1 (1:500, Wako Sobioda, France). The fragments were rinsed and then incubated with secondary antibodies: goat anti-Chicken IgY Alexa 647 and goat anti-rabbit IgG Alexa 488 (1:500, Molecular Probes, Invitrogen, Eugene, OR, United States) for 2 days at 4°C followed by incubation at room temperature for 1 h. The nuclear stain, 4′,6-diamidino-2-phenylindole (DAPI), was added to the incubating solution prior to washing steps.

The implant/retina ensemble was then rinsed and mounted, in permanent mounting medium (MM France, France), on a microscope slide, for viewing under an upright confocal microscope (FV1000, Olympus). DAPI counterstaining, AlexaFluor-488, and AlexaFluor-647 were used to be detected by excitation with a 405 nm laser diode, a 488 nm argon ion laser, and 635 nm laser diode lines, respectively.

### Microglial Count and Statistical Analysis

Confocal data was imported into Bitplane Imaris software for visualization and analysis of microglial cells in the entire retina using the Surface creation wizard. The Iba1 channel was selected and surfaces were generated from the signal using a set threshold to accurately visualize the source signal, which was then modeled by the software. Cells were verified by colocalization using the DAPI signal marking cell nuclei. False counts, duplicates as well as cells overlapping the boarder of the image were filtered before displaying the final result. Average cell volume was obtained by dividing counted cells by the total area generated from the signal source. Significance test was done using an unpaired two-tailed Welch’s *t*-test utilizing a *p* < 0.05 significance level.

## Results

### Microfabrication

**FIGURE 2:**
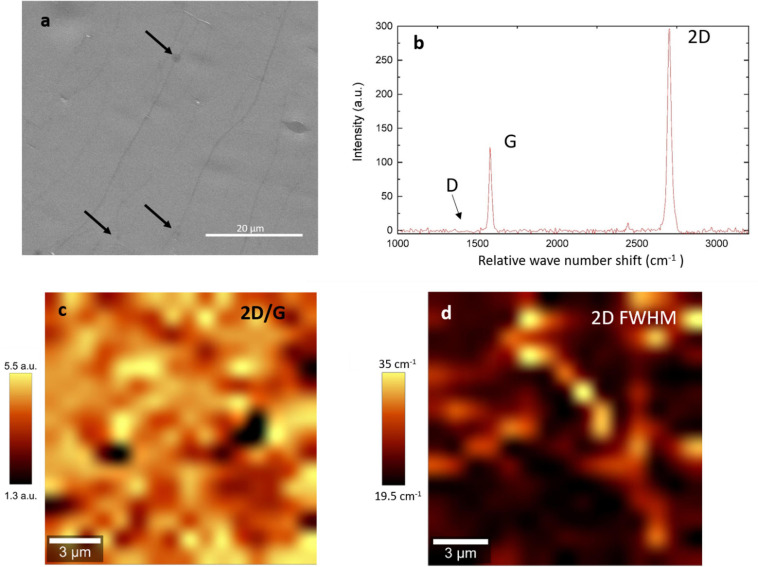
SEM and Raman spectroscopy of the single layer graphene **(a)** SEM of graphene on copper. The black arrows show the position of the second nucleation, **(b)** representative Raman spectrum, no D band can be observed, **(c)** 2D/G mapping of a 15 μm × 15 μm area, **(d)** FWHM mapping of a 15 μm × 15 μm area.

The layer graphene grown for this study were characterized on copper foil after the graphene growth and before their transfer to the polymer substrate using SEM and Raman spectroscopy. The SEM image (Figure 2a) shows that the graphene layer is closed. Additionally some small second nucleation can be distinguished every 50 μm. Figure 2b shows a representative Raman spectrum of the layer graphene. Figures 2c,d are mappings of 15 μm × 15 μm of the 2D/G ratio and 2D full width at half maximum (FWHM). The G (around 1590 cm^–1^) and 2D (around 2720 cm^–1^) bands of graphene can be clearly observed while the By contrast, the D (around 1350 cm^–1^) band cannot be distinguished suggesting that the layer has very few defects (Ferrari, 2007). Furthermore, the value of 2D/G ratio of around 4, and the 2D full width at half maximum (FWHM) of around 25 cm^–1^ combined to the good homogeneity of this characteristics over the mapped area. This provides proof of a good crystalline quality single graphene layer (Ferrari, 2007; Roscher et al., 2019) which has be shown to be an important factor for neural affinity (Veliev et al., 2016). Using the metallic tracks designed on the device extending up to the circular head of the device, the successful transfer of graphene on this implant head was verified by measuring the resistance between the tracks. The resistance measured for all the implants was contained between 0.5 and 1 kOhms instead of MOhms in the absence of graphene indicating that the two tracks were indeed connected by the deposited graphene.

### Implantation and *in vivo* Monitoring

**FIGURE 3:**
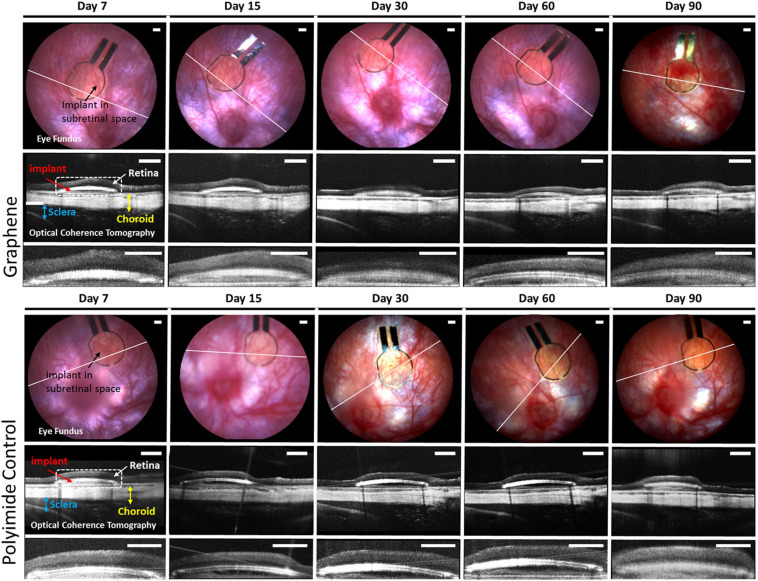
*In vivo* imaging of devices with graphene **(top set)** and devices without graphene **(bottom set)** at different specified time intervals. Each set includes eye fundus **(top row)**, OCT scans of device in the eye **(middle row)**, and a magnified image of OCT scan showing only the retina and device **(bottom row)**. Eye fundus provides evidence for the long-term stability of both types of devices up to 90 days. The white line indicates the location where the OCT scans were taken. The OCT scans shows the implant (red arrow) sandwiched between the retina (white arrow) and the choroid (yellow). Dotted white box is a zoom of each OCT scan showing in detail the retina and device. The magnified images of the OCT scans (bottom row) show the close contact of the retina and implant devices. Bottom row of each set is the zoom on the retina in contact with implant as indicated by dashed line of the OCT scan. All scale bars are 200 μm.

A total of 14 animals were used for this study. The animal weighed an average 348 ± 15 g at the time of implantation and 393 ± 25 g at time of euthanasia 3 months later. To verify the implant position, and in order to monitor any *in vivo* tissue inflammation, the eye fundus and OCT image of an optical retinal section was taken at specified intervals of 7, 15, 30, 60, and 90 days. Figure 3 illustrates an example of the *in vivo* monitoring by eye fundus and OCT scanning of an animal with a graphene implant and an animal with a control polyimide implant. It should be noted that eye fundus was taken during a transition from the Micron III to the Micron IV machine therefore displaying a transition in image results between day 60 and day 90 for the graphene images and between day 15 and day 30 for the polyimide control images. By eye fundus imaging, it can be seen that the implant was placed dorsal to the optic nerve and remained in the same location during the entire monitoring period. Additionally, no apparent delamination of the graphene, passivation layer, nor the metal contacts can be identified. The implant was confirmed to be in the subretinal space visualized by the large retinal vasculature above the implant, the white line indicates the area where the OCT image was taken.

Optical coherence tomography imaging provided a second confirmation of a subretinal implantation, showing that the implant was placed between the choroid and the retina. OCT images displayed in Figure 3 were averaged from five consecutive frames of the OCT scan at the location of the eye indicated by the white line in the eye fundus images. The OCT imaging demonstrated that retinal reattachment had occurred at day 7 with no subsequent retinal detachment until the end of the monitoring for both types of implants. Looking closely at the implant/retina OCT images (bottom row of Figure 3), the retina seems to be uniform and in close contact with the implant, indicating no inflammation. Supplementary Figure 1 shows the eye fundus and OCT scan of P23H rats at age of 9 and 12 months that had no implantation.

### Immunohistochemistry

**FIGURE 4:**
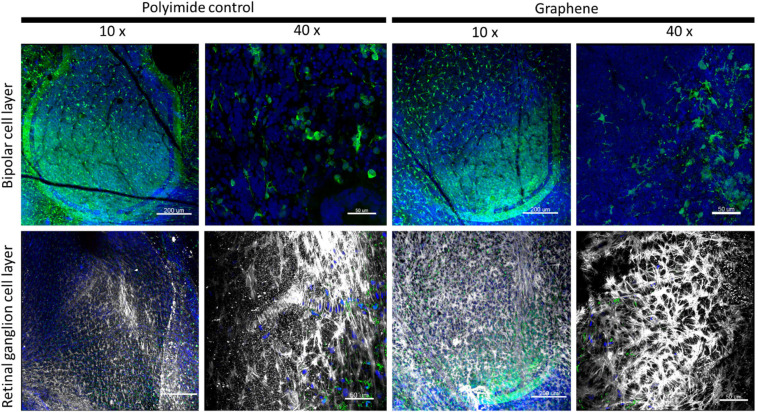
Low (10x) and high (40x) magnifications of retinal tissues at the graphene and polyimide implant interface. Images show the different immunolabeled cells: green Iba1-positive microglia, and white GFAP-positive Müller and astrocyte cells with blue DAPI-stained nuclei. Note the green autofluorescent polyimide ring of the implants at the 10x magnification in the bipolar layer of the polyimide control and at both layer for the graphene implant. Scale bars are 50 μm (10x) and 200 μm (40x).

**FIGURE 5:**
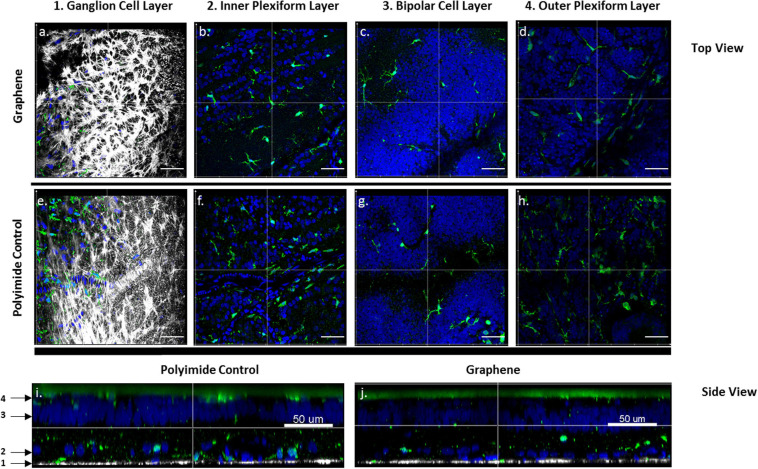
Microglial distributions within the retinal tissues at the graphene **(a–d,j)** and control polymer interface **(e–i)**. Confocal images of the retinal tissues showing Iba1-positive microglia cells (green) at different depths. **(a,e)** Optic fiber layer showing GFAP-positive Müller and astrocyte cells (white) and no microglial cells. Iba1-positive microglial cells with DAPI-stained nuclei (blue) at the junction between the inner plexiform layers (IPL) and the ganglion cell layer (GCL) **(b,f)**, in the inner nuclear layer (INL) **(c,g)** in the outer plexiform layer (OPL) **(d,h).** Confocal reconstruction of vertical sections for the retina above the control polymer implant **(i)** and the graphene implant **(j)** showing the different depths (1–4) for all above horizontal sections **(a–h)**. Scale bars represent 100 μm in **(a–h)** and 50 μm in **(i,j)**.

After the *in vivo* monitoring, inflammation at the cellular level was examined. The eye fragment containing the implant was fixed and treated to visualize glial cells. Histological examinations of the tissue indicated the well-preserved retina above the implant, as it can be seen at a 10x magnification in Figure 4. An immunolabelling was achieved to reveal the microglial cells (Iba1 antibody) and macroglial cells (GFAP antibody), while the DAPI staining revealed all cell nuclei within the retinal tissue above the implant. At the low magnification (10x), the implant could be seen below the retina by the green polyimide autofluorescence when imaging at the bipolar cell layer of both types of devices (Figure 4), and also when looking at the vertical retinal sections (Figures 5i,j). On the same field of view, the GFAP-macroglial cells were observed to remain at the surface of the retinal tissue. In both types of implants (Figure 4), the classic dense retinal network of astrocytes was visible at the low and high magnifications. Iba1-immunopositive microglial cells (green in Figure 4, 5) were seen in the tissue depth to form a more discrete network of cells among other cells revealed by DAPI-stained nuclei (blue in Figures 4, 5). The distribution of microglial cells could be discretely defined inside the tissue depth by serial optical confocal imaging allowing reconstruction of vertical sections showing the different layers of DAPI-stained nuclei (Figure 5). This cell type were observed in different horizontal levels within the retinal tissue in the inner plexiform layer (IPL) below the ganglion cell layer containing dispersed DAPI-stained nuclei and in the outer plexiform layer (OPL) just below the implant. The inner nuclear layer (INL) composed by dense DAPI-stained nuclei was devoid of microglial cells as in the normal tissue. Therefore, the microglial distribution appeared normal within the depth of the retina with no abnormal accumulation at the surface of the graphene implant direct contact.

### Statistical Analysis of Inflammatory Markers

**FIGURE 6:**
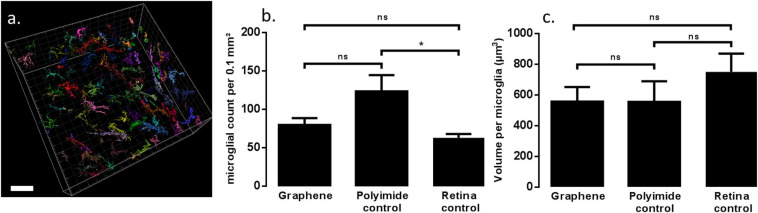
Modeling of microglia based on Iba1 marker on retinal tissue in contact with devices with graphene **(a)**. Each different color represent a different microglial. Scale bars are 50 μm. Graph **(b)** is the statistical analysis of microglial count comparing retinal tissue in contact with the two types of implant and retinal tissue from non-operated eyes. Graph **(c)** is the comparison of microglial volume between the three conditions. ns indicates *p* > 0.05, * indicates *p* ≤ 0.05.

To further quantify any potential retinal reactive state at the graphene implant interface, the microglial cells above the implants were counted. Figure 6a illustrates the strategy used for quantifying microglial cells within the retinal volume with a 0.1 mm^2^ base area. Using Iba1 data from confocal imaging, microglial cells could be reconstituted within the investigated volume for a semi-automated counting. Individual cells were quantified for both graphene implants and control polyimide implants. A quantification on non-operated P23H rat eyes at the same age was also added for comparison to the degenerated retina. This quantification showed that the number of microglial cells within the retina was significantly greater in the presence of the control polyimide implant (123.6 ± 21.1, *n* = 5) than in the control non-operated eyes (67.0 ± 8.4, *n* = 7, *p* = 0.044). This could be expected from the surgical stress and the implant presence (Figure 6b). However, this difference was reduced for implants consisting graphene (79.86 ± 8.8, *n* = 7) with respect to the retina from non-operated eyes with no significant difference between the two. All differences between the graphene implant with either the polymer implants (*p* = 0.110) and the control P23H retina (*p* = 0.131) were not statistically significant. The results by microglial count signify that graphene is more biocompatible than the polymer implant.

In pathological conditions, microglial cells are known to change from a star-like shape with long processes indicating a relaxed state to a more amoeboid morphology. This change in state alters the cell body volume, which can be quantified through the Iba1-immunolabeled microglial cells. In non-operated eyes, the microglial cell volume (749.9 ± 118.8 μm^3^) was greater than that measured for the graphene implant (556.2 ± 251.8 μm^3^) and for the control polymer implants (554.8 ± 299.9 μm^3^). Though there are no significant differences in microglial volume between non-operated eye and operated eye (*p* < 0.5), the average volume in the graphene implant and polymer implant is similar to each other and smaller than non-operated tissue. The results signify that graphene does not impact the biocompatibility of polyimide and SU-8 polymers.

## Discussion

Graphene is a new carbon-based material with a large water window providing exceptional electrical performances for neurophysiological recording and capable charge injection for neuronal stimulation. Despite this, free carbon-nanotubes were found to present some toxicity issues (Yan et al., 2019). Therefore, it is necessary to determine whether a carbon-based electrode tethered to a device would present toxicity to nervous tissue. Previous cell culture studies have already demonstrated the high biocompatibility of CVD graphene bounded to a hard material (glass) for retinal neurons (Bendali et al., 2013) as well as investigated its effect on peripheral neurons (Convertino et al., 2018). Additionally, CVD graphene coated onto intracortical probes that were chronically implanted in mice cortex showed that that the presences of graphene had reduced the local density of astrocytes and microglia at the implanted site (Bourrier et al., 2019). Following graphene transfer on soft biocompatible polymer material, the *in vivo* biocompatibility of graphene in the retina was shown here. The retina represents a relatively accessible location to the central nervous system and could bypass complication of placing materials directly on the brain. These results are important for further use of graphene in retinal prostheses for restoring vision. However, the results have wider relevance for applications in any other part of the central nervous system.

In the context of visual restoration, the study was performed in the blind homozygous P23H rat, which is widely used as a model of retinitis pigmentosa due to a complete degeneration of photoreceptors occurring prior to 9 months (Pinilla et al., 2005; Orhan et al., 2015). The 3 months *in vivo* follow-up showed a high stability of the large area of graphene placed between flexible polyimide and SU-8 substrate without any delamination. Generally, retinal implant arrays contain microelectrodes (less than 100 μm in diameter) for electrical stimulation. Here, a worst-case scenario was assessed by using a 1 mm diameter electrode, which represents the largest possible graphene-retinal interface without increasing the dimensions of the device. The graphene electrode was connected to two metal contacts used to verify the presence of graphene. However, the lack of functional electrode in these devices did not allow for the assessment of tissue damage and inflammation that could result from electrical stimulation.

Functional devices require a tail relayed to an external headstage connector, which is not the case here. However, such complete systems introduce an additional mechanical constraint within a moving eye could lead to potential shear stress at the electrode array which could result in an inflammation. Such functional devices had been for instance produced with titanium–platinum or diamond electrodes for monitoring the evolution of electrode impedance over time (Linderholm et al., 2013; Hébert et al., 2016). Eye fundus examination and OCT imaging confirmed the absence of abnormal situations (edema and hemorrhage, Linderholm et al., 2013) during the *in vivo* period. This *in vivo* monitoring further demonstrated that graphene remained in tight contact with the retinal tissue for the whole implantation period.

Microglial cells act in the retina and more generally in the central nervous system as resident macrophages assessing cell function and any intrusion. Biocompatibility of various materials could be investigated by assessing the reactive microglia status as indicated by microglial proliferation and amoeboid transformation into macrophages. In the degenerating retina, microglial cells are expected to be already in a reactive state. We therefore decided to use the Iba1 marker to label both reactive and relaxed microglial cells in the retina to visualize the inflammatory response at the implant position. Assessing cell volume gave an indication on morphology, which indirectly relates to the active/non-reactive state of the tissue. Although surgery and the presences of a retinal implant induced a change in microglial features, microglial count in the retina in contact with polymer implants was significantly greater than in non-operated eyes. However, there was no significant difference between that of graphene implants and non-operated eyes. Additionally, average microglial volume showed no significant differences between graphene and control implants made with biocompatible polymers. In addition, there were no significant differences between both implant and non-operated eyes. These findings are consistent with a high biocompatibility of graphene-based electrodes as previously suggested with *in vitro* studies (Bendali et al., 2013; Convertino et al., 2018), and *in vivo* studies (Bourrier et al., 2019). However, future works should assess neuronal function to demonstrate further the biocompatibility of the graphene material although this assessment is particularly difficult in blind animals with a flat electroretinogram (Pinilla et al., 2005; Orhan et al., 2015).

## Conclusion

Assessing *in vivo* material biocompatibility is an essential step in developing new technologies for neuronal recording and stimulation in electronic devices. This study focused on CVD graphene material and its potential use in active electrodes. The study demonstrates that the presences of graphene induces a lesser inflammation quantified by microglial labeling compared to biocompatible polymers. Graphene can therefore be further investigated for use in electrodes for retinal prostheses or more generally any electronic device for recording and stimulation of the central nervous system. Further studies will assess more developed graphene structures on chronic devices to compare biocompatibility, safety and stimulation capabilities between classic materials and graphene-based electrodes.

## Data Availability Statement

The raw data supporting the conclusions of this article will be made available by the authors, without undue reservation.

## Ethics Statement

The animal study was reviewed and approved by Charles Darwin No5 Ethics Committee in Animal Experimentation (agreement #15219). Written informed consent was obtained from the owners for the participation of their animals in this study.

## Author Contributions

DN: conceptualization, methodology, investigation, writing-original draft, formal analysis, and visualization. MV: methodology, writing-original draft, resources, investigation, and formal analysis. JD: methodology, resources, investigation, and writing-review and editing. LB: investigation, formal analysis, and writing-review and editing. XI: validation, investigation, and writing-original draft. JC and EC: validation, writing-review and editing. JB: investigation, validation, data curation, and writing-review and editing. JG: conceptualization, supervision, writing-review and editing, and funding acquisition. CH: conceptualization, investigation, supervision, visualization, validation, writing-original draft, and writing-review and editing. SP: conceptualization, supervision, funding acquisition, writing-original draft, and writing-review and editing. All authors: contributed to the article and approved the submitted version.

## Conflict of Interest

SP is a funder and shareholder of Pixium Vision. The remaining authors declare that the research was conducted in the absence of any commercial or financial relationships that could be construed as a potential conflict of interest.

## Supplementary Material

Supplementary Figure 1 Typical imaging of eye fundus and OCT scan of non-implanted P23H rat at 9 and 12 months of age. The white line on the eye fundus indicates the location where the OCT scans were taken. The OCT scans shows the retina (white arrow), the choroid (yellow) and the sclera (blue). Dotted white box is a zoom of each OCT scan showing in detail the retina. All scale bars are 200 μm.
