# Supplementary figures and images for: Novel Graphene Electrode for Retinal Implants: An in vivo Biocompatibility Study
Source: Front Neurosci. 2021 Mar 4;15:615256. doi: 10.3389/fnins.2021.615256 (PMC7969870; doi:10.3389/fnins.2021.615256)

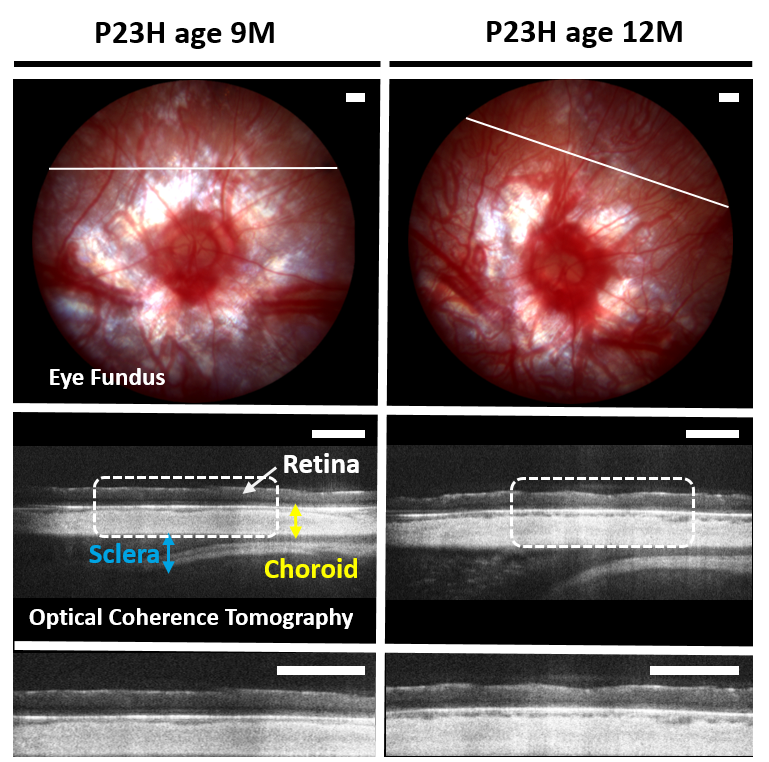

Supplement: Supplementary Figure 1 — Typical imaging of eye fundus and OCT scan of non-implanted P23H rat at 9 and 12 months of age. The white line on the eye fundus indicates the location where the OCT scans were taken. The OCT scans shows the retina (white arrow), the choroid (yellow) and the sclera (blue). Dotted white box is a zoom of each OCT scan showing in detail the retina. All scale bars are 200 μm. [file Image_1.TIF]
